# Quercetin-3-Rutinoside Blocks the Disassembly of Cholera Toxin by Protein Disulfide Isomerase

**DOI:** 10.3390/toxins11080458

**Published:** 2019-08-04

**Authors:** Jessica Guyette, Patrick Cherubin, Albert Serrano, Michael Taylor, Faisal Abedin, Morgan O’Donnell, Helen Burress, Suren A. Tatulian, Ken Teter

**Affiliations:** 1 Burnett School of Biomedical Sciences, College of Medicine, University of Central Florida, Orlando, FL 32816, USA; 2 Department of Physics, College of Sciences, University of Central Florida, Orlando, FL 32816, USA

**Keywords:** AB toxin, cholera toxin, inhibitor, polyphenol, protein disulfide isomerase, ricin, rutin hydrate

## Abstract

Protein disulfide isomerase (PDI) is mainly located in the endoplasmic reticulum (ER) but is also secreted into the bloodstream where its oxidoreductase activity is involved with thrombus formation. Quercetin-3-rutinoside (Q3R) blocks this activity, but its inhibitory mechanism against PDI is not fully understood. Here, we examined the potential inhibitory effect of Q3R on another process that requires PDI: disassembly of the multimeric cholera toxin (CT). In the ER, PDI physically displaces the reduced CTA1 subunit from its non-covalent assembly in the CT holotoxin. This is followed by CTA1 dislocation from the ER to the cytosol where the toxin interacts with its G protein target for a cytopathic effect. Q3R blocked the conformational change in PDI that accompanies its binding to CTA1, which, in turn, prevented PDI from displacing CTA1 from its holotoxin and generated a toxin-resistant phenotype. Other steps of the CT intoxication process were not affected by Q3R, including PDI binding to CTA1 and CT reduction by PDI. Additional experiments with the B chain of ricin toxin found that Q3R could also disrupt PDI function through the loss of substrate binding. Q3R can thus inhibit PDI function through distinct mechanisms in a substrate-dependent manner.

## 1. Introduction

Cholera toxin (CT) is an AB_5_-type protein toxin that contains an enzymatic A1 subunit, an A2 linker, and a cell-binding B homopentamer [1]. The intact holotoxin is released into the extracellular milieu by *Vibrio cholerae* and binds to GM1 gangliosides on the surface of a target cell. This results in toxin endocytosis through a mechanism involving lipid rafts [2]. The internalized toxin then moves by retrograde vesicular transport from the endosomes, through the Golgi apparatus, and to the endoplasmic reticulum (ER) [3]. A disulfide bridge linking CTA1 to CTA2 is reduced in the ER [4,5]. This is followed by the chaperone-assisted separation of CTA1 from CTA2/CTB_5_ [6,7]. The free CTA1 subunit spontaneously shifts to a disordered conformation that promotes its export to the cytosol through the mechanism of ER-associated degradation [8,9,10]. In the cytosol, CTA1 interacts with host factors to regain a folded, functional conformation [11,12,13,14]. It then elevates intracellular cAMP through an ADP-ribosylation reaction that locks its Gsα target in a constitutively active state. The resulting dysregulation of signal transduction leads to the opening of chloride channels at the apical face of the intestinal epithelium and chloride efflux into the gut. The osmotic movement of water that follows this chloride efflux produces the rice-water stools characteristic of cholera [15,16].

The cytopathic activity of CT requires an interaction with protein disulfide isomerase (PDI). The CTA1/CTA2 disulfide bond can be reduced by PDI, but this can also occur at the resident redox state of the ER [4] and does not itself result in holotoxin disassembly [17]. Instead, the essential function of PDI in CT intoxication involves the physical displacement of reduced CTA1 from its non-covalent assembly in the CT holotoxin. This results from the partial unfolding of PDI that occurs upon contact with CTA1: the expanded size of disordered PDI acts as a wedge to separate CTA1 from CTA2/CTB_5_ [18]. Only PDI can disassemble CT [18], so PDI-deficient cells are resistant to CT [6].

PDI has a modular **abb’xa’c** structure that consists of two active thioredoxin domains (**a** and **a’**), two inactive thioredoxin domains (**b** and **b’**), an **x** linker, and a short acidic **c** region [19]. The **a** and **a’** domains allow PDI to function as an oxidoreductase, but this enzymatic activity is not required for PDI to act as a chaperone [20,21]. The **b** and **b’** domains at the base of the U-shaped protein serve as the main sites of substrate binding [22,23,24]. The **x** linker is an unstructured segment that contributes to the conformational flexibility of PDI, while the **c** region contains a KDEL tag that retains PDI in the ER [25]. Interactions at one domain of PDI influence the structure/function of other PDI domains [24,25,26,27,28]. PDI also undergoes redox-dependent conformational changes that can influence its interactions with select substrates [29]. For example, only the reduced form of PDI binds to CTA1 [6,7].

PDI mainly resides in the ER, but it is also found at other locations [30,31,32,33]. Several cell types secrete PDI into the bloodstream where its oxidoreductase activity promotes thrombus formation [34,35,36]. This activity is blocked by quercetin-3-rutinoside (Q3R), a flavanol that binds to the **b’x** region of PDI [37,38]. Q3R was identified from a high-throughput screen for PDI inhibitors that could be used as potential therapeutics to prevent blood clots [39], and it does not affect related oxidoreductases such as ERp57 or ERp72 [38,39,40]. However, its inhibitory mechanism against PDI is not fully understood.

Here, we report that Q3R blocked the PDI conformational change that occurs when it binds to CTA1. This prevented disassembly of the CT holotoxin and CT intoxication of cultured cells. Other steps of the CT intoxication process were not affected by Q3R, including PDI binding to CTA1 and reduction of the CTA1/CTA2 disulfide bond by PDI. We also identified a second, distinct mechanism for the inhibitory action of Q3R: it prevented PDI from binding to the B subunit of ricin, an AB-type plant toxin. Our observations document two separate mechanisms for the inhibitory effect of Q3R on PDI function and establish a potential therapeutic role for Q3R in the treatment of cholera.

## 2. Results

### 2.1. Q3R Protects Cultured Cells from CT

**Figure 1:**
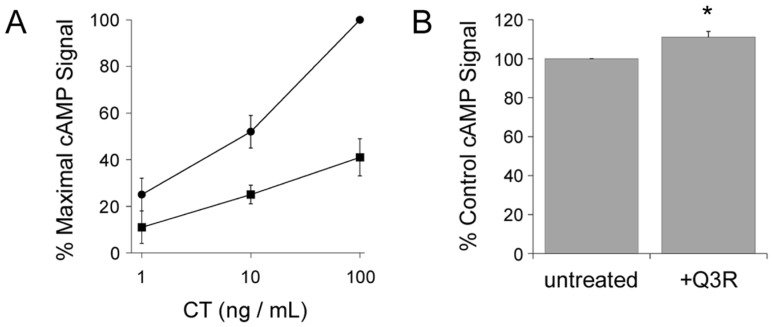
Quercetin-3-rutinoside (Q3R) inhibits the cytopathic activity of exogenously applied cholera toxin (CT) but not ER-localized CTA1. (**A**) CHO cells were exposed to various concentrations of CT for 2 h in the absence (circles) or presence (squares) of 100 µM Q3R before intracellular cAMP levels were quantified. Background-subtracted data (means ± SEMs; *n* = 5) are presented as percentages of the maximal cAMP response from cells exposed to 100 ng/mL of CT in the absence of drug treatment. (**B**) CHO cells transfected with a plasmid encoding an ER-targeted CTA1 construct were chased for 4 h in the absence or presence of 100 µM Q3R before intracellular cAMP levels were quantified. Background-subtracted data (means ± SEMs; *n* = 4) are presented as percentages of the maximal cAMP response from transfected cells chased in the absence of Q3R (untreated). The asterisk denotes a statistically significant difference (*p* = 0.0142, Student’s *t* test) from the cAMP response of untreated cells.

We hypothesized Q3R would block the cytopathic activity of CT through its inhibitory action on PDI. To test this prediction, cAMP levels were quantified from CHO cells incubated with CT for 2 h in the absence or presence of Q3R. As shown in Figure 1A, Q3R-treated cells were protected from the cytopathic activity of CT. In contrast, Q3R did not inhibit the cAMP response generated from cells expressing an ER-localized CTA1 construct (Figure 1B). Transfection with the pCDNA3.1/ssCTA1 plasmid results in co-translational insertion of CTA1 into the ER lumen, proteolytic cleavage of its N-terminal ER-targeting signal sequence (ss), and dislocation of CTA1 back into the cytosol where it elicits a cAMP response beginning 2 h post-transfection [41]. We found the cAMP response from Q3R-treated cells was 11% greater than the response from untreated cells that were also expressing CTA1. The basis for this mild sensitization remains to be determined, but the result clearly shows that Q3R does not block (i) the ER-to-cytosol export of free CTA1 or (ii) the enzymatic activity of cytosolic CTA1. The inhibitory effect of Q3R on CT intoxication thus occurs at a step preceding translocation of free CTA1 to the cytosol.

### 2.2. Q3R Does Not Prevent PDI from Binding or Reducing CTA1

**Figure 2:**
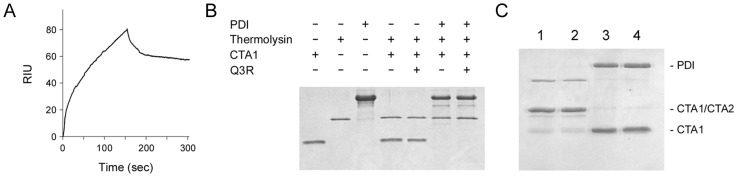
Q3R does not affect protein disulfide isomerase (PDI) binding to CTA1 or reduction of the CTA1/CTA2 disulfide bond. (**A**) Q3R-treated PDI was perfused at 25 °C over an SPR sensor coated with CTA1. PDI was removed from the perfusion buffer after 150 s. One of two representative experiments is shown. (**B**) The indicated combinations of PDI, CTA1, and Q3R were incubated at 30 °C for 1 h before placement in an ice bath. Thermolysin was then added to the specified samples for a 1 h incubation at 4 °C before SDS-PAGE with Coomassie stain. One of two representative experiments is shown. (**C**) A purified, disulfide-linked CTA1/CTA2 heterodimer was incubated for 30 min at 37 °C with no additions (lane 1); Q3R (lane 2); pre-reduced PDI (lane 3); or Q3R and pre-reduced PDI (lane 4). Samples were then resolved by non-reducing SDS-PAGE with Coomassie stain. One of two representative experiments is shown.

Surface plasmon resonance (SPR) was used to examine the impact of Q3R on PDI binding to CTA1 (Figure 2A). For this experiment, CTA1 was coupled to a SPR sensor. A baseline measurement corresponding to the mass of sensor-bound CTA1 was set at 0 refractive index units (RIU), and Q3R-treated PDI was then added to the perfusion buffer. The elevated signal following this injection indicated Q3R-treated PDI had bound to CTA1, thereby adding mass to the sensor surface and altering the RIU. The pattern of binding was similar to what was previously reported for untreated PDI [6]. Thus, Q3R did not disrupt the physical association between PDI and CTA1.

Contact with PDI at 30 °C will shift CTA1 to a protease-sensitive state [7]. A protease sensitivity assay was performed to examine the impact of Q3R on this interaction (Figure 2B). Following an established protocol [42], samples of CTA1 were incubated for 1 h at 30 °C with various combinations of PDI and Q3R. All samples were then placed on ice and incubated with the thermolysin protease for 1 h at 4 °C before visualization by SDS-PAGE and Coomassie stain. CTA1 remained in a protease-resistant state when exposed to Q3R alone, but, upon exposure to either PDI or Q3R-treated PDI, it shifted to a protease-sensitive conformation that resulted in its complete degradation. Thus, Q3R-treated PDI can still bind to CTA1 and convert the toxin to a protease-sensitive state.

Q3R blocks the PDI-mediated reduction of insulin [37,38,39,40,43], but it did not inhibit the ability of PDI to reduce the CTA1/CTA2 disulfide bond and did not itself directly reduce the CTA1/CTA2 disulfide bond (Figure 2C). Our reduction and protease sensitivity assays used a 150-fold molar excess of Q3R over PDI, as previous studies have reported inhibitory effects on the oxidoreductase activity of PDI when Q3R was present at a 40, 70, or 100-fold molar excess over PDI [37,38,39,43]. Our SPR binding assay used an even greater molar excess (3000-fold) of Q3R over PDI, yet we still did not see an inhibitory effect on PDI-CT interactions in this experiment. As such, the different outcomes of Q3R treatment on PDI interactions with insulin vs. CTA1 could not be attributed to different drug concentrations. Instead, Q3R appears to have substrate-dependent inhibitory effects on the function of PDI.

### 2.3. Q3R Disrupts PDI-Driven Disassembly of the CT Holotoxin

**Figure 3:**
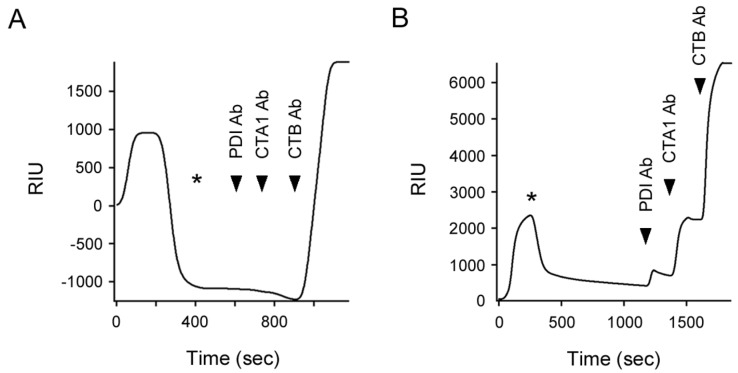
Q3R inhibits PDI-mediated disassembly of the CT holotoxin. After applying CT to a GM1-coated SPR sensor, a baseline measurement (0 RIU) recorded the mass of the sensor-bound holotoxin. (**A**) PDI or (**B**) Q3R-treated PDI was then perfused over the slide at 37 °C, beginning at time 0. Asterisks indicate when PDI was removed from the perfusion buffer. The arrowheads represent sequential additions of PDI, CTA1, and CTB antibodies. One of four representative experiments using a 150:1 molar ratio of Q3R:PDI is shown in panel B.

Our collective data indicated Q3R does not block PDI binding to CTA1, the PDI-induced shift in CTA1 protease sensitivity, or reduction of the CTA1/CTA2 disulfide bond by PDI. We next examined the effect of Q3R on disassembly of the CT holotoxin by PDI (Figure 3). For this experiment, CT was appended to a GM1-coated SPR sensor slide. A baseline measurement corresponding to the mass of the captured holotoxin was then set at 0 RIU. Subsequent addition of PDI to the perfusion buffer resulted in an elevated RIU signal indicative of PDI binding to CT. In the absence of Q3R (Figure 3A), the rise in RIU was followed by a rapid drop in signal to a point below the baseline value corresponding to the mass of the CT holotoxin. This drop occurred despite the continued presence of PDI in the perfusion buffer and was attributed to the PDI-mediated release of CTA1 from the sensor-bound CTA2/CTB_5_ complex. Both PDI and its displaced CTA1 binding partner would be removed from the sensor by the flow of the perfusion buffer, thus lowering the RIU below its initial baseline value. After removing PDI from the perfusion buffer, sequential antibody injections confirmed our interpretation: no signal was obtained from the PDI and CTA1 antibodies, whereas the CTB antibody produced a positive result. This result was consistent with previous reports that used SPR to monitor the PDI-driven disassembly of CT [6,18].

As with untreated PDI, injection of Q3R-treated PDI into the perfusion buffer produced an elevated RIU (Figure 3B). However, no loss of signal occurred while Q3R-treated PDI was in the perfusion buffer. The RIU only dropped when Q3R-treated PDI was removed from the perfusion buffer, which indicated PDI had been released from the intact CT holotoxin. Yet the signal remained above the initial baseline measurement and never dropped below 0 RIU. It thus appeared that some PDI remained associated with the sensor-bound holotoxin for an extended time. This interpretation was confirmed by the positive signals generated from each sequential perfusion of anti-PDI, anti-CTA1, and anti-CTB antibodies over the sensor. Q3R-treated PDI could thus bind to the CT holotoxin but could not efficiently separate CTA1 from CTA2/CTB_5_.

### 2.4. Q3R Blocks the Conformational Change in PDI that Occurs with Its Binding to CTA1

**Figure 4:**
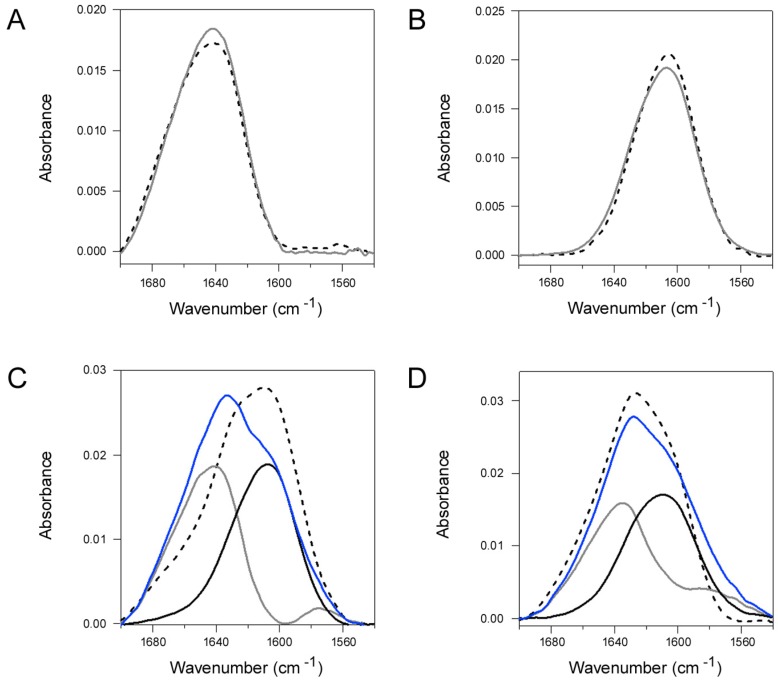
Q3R blocks the toxin-induced conformational change in PDI. (A-B) FTIR spectra were recorded for (**A**) PDI and (**B**) ^13^C-labeled CTA1 alone (dashed lines) or in the presence of Q3R (grey solid lines). (**C**) The measured FTIR spectra of PDI alone (grey line) and ^13^C-labeled CTA1 alone (black line) were used to generate a predicted spectrum for the combination of PDI and ^13^C-labeled CTA1 (blue line). The dashed line presents the measured FTIR spectrum for the combination of PDI and ^13^C-labeled CTA1. (**D**) The measured FTIR spectra of Q3R-treated PDI (grey line) and Q3R-treated, ^13^C-labeled CTA1 (black line) were used to generate a predicted spectrum for the combination of PDI and ^13^C-labeled CTA1 in the presence of Q3R (blue line). The dashed line presents the measured FTIR spectrum for the combination of PDI and ^13^C-labeled CTA1 in the presence of Q3R. For all applicable experiments, Q3R was present at a 150-fold molar excess over PDI and a 55-fold molar excess over CTA1. One of two representative experiments conducted at 25 °C is shown for each panel.

PDI assumes a compact, rigid structure in the presence of Q3R [38]. This could affect the structural change in PDI that is required for disassembly of the CT holotoxin. We examined this possibility with Fourier transform-infrared (FTIR) spectroscopy, a technique that can detect changes in the secondary and tertiary structures of proteins [44,45]. As seen in Figure 4A, the spectrum of PDI (dashed line) did not shift or change shape in the presence of a 150-fold molar excess of Q3R (grey solid line). This indicated that Q3R did not alter the secondary or tertiary structure of PDI. Likewise, Q3R did not affect the overall secondary structure of CTA1: both untreated (dashed line) and Q3R-treated (grey solid line) samples of CTA1 produced nearly identical FTIR spectra (Figure 4B).

We next documented the impact of CTA1 binding on the structure of PDI (Figure 4C) and examined whether Q3R affected the toxin-induced structural change in PDI (Figure 4D). Uniformly ^13^C-labeled CTA1 was used for these experiments in order to distinguish the FTIR spectrum for CTA1 from the spectrum for PDI. As shown in Figure 4C,D, the spectrum of uniformly ^13^C-labeled CTA1 (black lines) exhibited ~40 cm^−1^ downshift in comparison to the spectrum for unlabeled PDI (grey lines), an expected effect of ^13^C-labeling. Adding the spectra of the two individual proteins produced a predicted spectrum for uniformly ^13^C-labeled CTA1 and PDI together (blue line). Remarkably, the measured spectrum for the actual combination of the two proteins in the absence of Q3R displayed a different line-shape and was significantly downshifted from the predicted spectrum (Figure 4C, dashed line). The major spectral downshift in the 1680–1640 cm^−1^ region reflects conformational change in PDI. The most straightforward interpretation of this result is the opening of PDI tertiary structure in the presence of CTA1. This would expose more PDI amino acids to D_2_O, allowing more efficient amide hydrogen/deuterium exchange with a consequent amide I spectral downshift for PDI. In contrast, no such shift was observed between the predicted (blue line) and measured (dashed line) spectra for the combination of uniformly ^13^C-labeled CTA1 and unlabeled PDI in the presence of Q3R (Figure 4D). This demonstrated that Q3R blocks the CTA1-induced transition of PDI to a more disordered conformation.

A 150:1 molar ratio of Q3R:PDI was sufficient to block the CTA1-induced conformational change in PDI (Figure 4D) and the disassembly of CT by PDI (Figure 3B), but it did not block PDI binding to CTA1 (Figure 2B) or PDI reduction of the CTA1/CTA2 disulfide bond (Figure 2C). Q3R thus had a specific disruptive effect on a subset of PDI-CT interactions. We next examined whether Q3R could also inhibit PDI interactions with ricin, another ER-translocating AB toxin.

### 2.5. Q3R Inhibits PDI Binding to the B Chain of Ricin.

**Figure 5:**
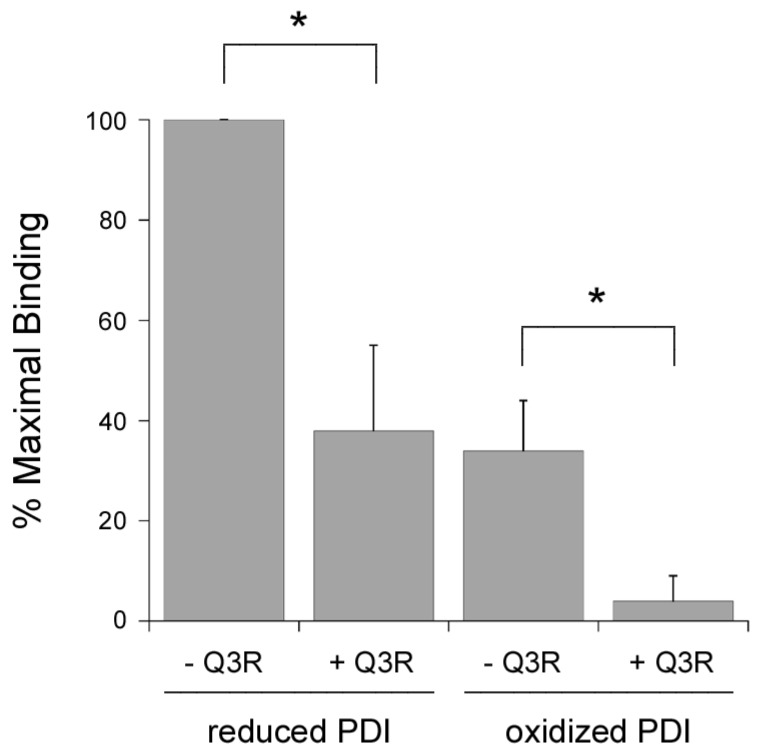
Q3R blocks PDI binding to RTB. An ELISA plate coated with RTB was exposed to reduced PDI (+ 1 mM DTT) or oxidized PDI in the absence or presence of Q3R as indicated. A 150:1 molar ratio of Q3R:PDI was used for the experiment. Data represent the averages ± standard deviations of three independent experiments with four replicate wells per condition. The strongest signal in each experiment (i.e., reduced PDI - Q3R) was set at 100% binding, and all other results were expressed relative to this value. Asterisks indicate statistically significant differences (*p* < 0.01, Student’s *t* test).

Q3R was originally identified as an inhibitor that blocked the PDI-mediated reduction of insulin [39]. We found that Q3R also blocked the PDI-mediated reduction of ricin (data not shown), a toxin that links its A and B subunits through a disulfide bond that can be reduced after PDI binding to the ricin toxin B subunit (RTB) [46,47,48]. The inhibitory effect of Q3R on ricin reduction could result from the disruption of PDI oxidoreductase activity or from a block of substrate binding. To further examine these possibilities, we used an ELISA-based assay to monitor the binding of Q3R-treated PDI to RTB (Figure 5). RTB was captured on an ELISA plate coated with asialofetuin II, a receptor for ricin [49]. PDI was then added to the plate for 1 h at 4 °C. After washing, the retained pool of PDI was detected with an anti-PDI primary antibody and HRP-conjugated secondary antibody. We found that PDI could bind to RTB in both the absence and presence of DTT, although reduced PDI (+ DTT) produced a stronger signal than oxidized PDI. The binding of both reduced and oxidized PDI to RTB was inhibited by a 150-fold molar excess of Q3R, with ~60% inhibition of binding for reduced PDI and a near-complete inhibition of binding for oxidized PDI. The same or greater molar ratio of Q3R:PDI had no effect on PDI binding to CTA1 (Figure 2A,B). Thus, Q3R can inhibit PDI through at least two independent mechanisms: it blocks the conformational change in PDI that is required for CT disassembly, and it blocks PDI binding to RTB. These results demonstrate Q3R can disrupt PDI function through distinct mechanisms in a substrate-dependent manner.

## 3. Discussion

Polyphenolic compounds such as Q3R frequently exhibit anti-toxin properties [50,51,52]. The inhibitory mechanisms are often unknown, but Q3R is an established inhibitor of PDI—a key protein in the CT intoxication process [6]. Here, we provide a molecular basis for the disruptive effect of Q3R on the interaction between PDI and CT. Our work also demonstrates Q3R can affect PDI through at least two distinct mechanisms.

We have proposed the partial unfolding of PDI allows it to dislodge CTA1 from its non-covalent assembly in the CT holotoxin: the expanded size of disordered PDI could push between CTA1 and CTA2/CTB_5_, acting as a wedge for the release of reduced CTA1 from its holotoxin. Conditions that stabilize the structure of PDI thus block CT disassembly by PDI [18]. Q3R likewise blocked both the CTA1-induced disordering of PDI structure and CT disassembly by PDI. Our work with Q3R thus provides additional evidence for the “wedge” model of toxin disassembly. Other steps of the CT intoxication process were not inhibited by Q3R, including PDI binding to CTA1, reduction of the CTA1/CTA2 disulfide bond, CTA1 dislocation to the cytosol, and CTA1 activity in the cytosol. We therefore conclude that Q3R generates a CT-resistant phenotype through a specific mechanism involving the stabilization of PDI and resulting inhibition of PDI-driven toxin disassembly.

Q3R causes PDI to assume a compact conformation with limited flexibility [38]. This effect likely explains why CTA1 cannot induce the conformational change in Q3R-treated PDI that is required for CT disassembly. However, it is unknown how the PDI-mediated reduction of insulin is disrupted by Q3R—an inhibition of substrate binding and an inhibition of PDI oxidoreductase activity are both options. Q3R did not affect either of these events for PDI-CTA1 interactions, but it did block PDI binding to RTB with a consequent inhibition of ricin reduction by PDI. Q3R can thus inhibit, in a substrate-dependent manner, either substrate binding to PDI or the substrate-induced unfolding of PDI. A direct inhibition of PDI oxidoreductase activity against insulin remains a possibility as well. Our collective results thus provide additional insight into the inhibitory action of Q3R that could possibly be used for therapeutic purposes to disrupt cellular events that require PDI activity.

Q3R occupies the **b’x** region of PDI [38], which is also the major substrate binding site in PDI [22,23,24]. Occlusion of the **b’x** region by Q3R could thus inhibit PDI binding to RTB and, possibly, insulin. However, PDI could still bind to CTA1 in the presence of Q3R. A site other than the **b’x** region may therefore represent the major point of contact between PDI and CTA1. This would be unusual for PDI-substrate interactions, but the interplay between PDI and CTA1 already has several atypical characteristics: binding of reduced but not oxidized PDI to CTA1; PDI binding to folded but not unfolded CTA1; and the partial unfolding of PDI upon contact with CTA1 [6,18]. Further studies will be required to test the hypothesis, derived from our current studies, that the **b’x** region is not involved with PDI binding to CTA1.

Disordered PDI remains bound to CTA1; it is the thermal unfolding of free CTA1 that leads to the release of PDI [6]. Thus, for its interaction with CTA1, discrete regions of PDI are likely involved with substrate binding and substrate-induced unfolding. Our results with Q3R-treated PDI are consistent with this possibility, as Q3R blocked the CTA1-induced disordering of PDI but not PDI binding to CTA1. The stabilization of PDI by a compound that occupies the **b’x** region of PDI suggests an essential role for **b’x** in the CTA1-induced disordering of PDI. This would be consistent with the influence of the **b’x** region on the conformational flexibility of PDI [25] as well as the compaction of PDI structure that is induced by Q3R binding to **b’x** [38].

Q3R is considered a promising antithrombotic agent because of its inhibitory action on extracellular PDI [53], but its mode of action is unknown. Here, we documented two distinct inhibitory effects for Q3R: it blocks the CTA1-induced disordering of PDI that is required for CT disassembly, and it prevents PDI binding to RTB. Our work provides insight into the molecular basis for the inhibitory action of Q3R and suggests Q3R could be developed as a therapeutic for cholera.

## 4. Materials and Methods

### 4.1. Materials

Q3R, asialofetuin II, CT, and CTA (i.e., the purified CTA1/CTA2 heterodimer) were purchased from Sigma-Aldrich (St. Louis, MO, USA). CTA1 with a C-terminal His_6_ epitope tag was purified by metal affinity chromatography as previously described [9]. RTB was provided by BEI resources (Manassas, VA, USA).

### 4.2. Purification of Recombinant PDI with an N-Terminal His_6_ Tag

*Escherichia coli* strain BL21(DE)pLysS transformed with the pOLR130 plasmid [54] was grown in 1 L Luria broth with 100 μg/mL of ampicillin and induced at an OD_600_ of 0.6 with 1 mM ITPG for 4 h at 37 °C. Induced cultures were spun for 20 min at 6000 rpm and 4 °C before freezing the cell pellet at −80 °C. Pellets were resuspended in lysis buffer (100 μg/mL lysozyme; 1% deoxycholate; 0.1% Triton X-100; 20 mM sodium borate buffer pH 7.0; 300 mM sodium chloride) and subjected to sonication. The cell lysate was spun at 12,000 × *g* for 20 min at 4 °C, and the resulting supernatant was used for affinity chromatography.

TALON beads (Clontech, Mountain View, CA, USA) were washed three times with extraction buffer (20 mM sodium borate buffer pH 7.0; 300 mM sodium chloride) and spun down for 2 min at 700× *g*. Once washed, the sonicated, clarified cell lysate was added to the beads and rotated for 45 min at room temperature. The PDI-bound beads were then washed 3 times in wash buffer (20 mM sodium borate buffer pH 7.0; 600 mM sodium chloride; 0.1% Triton X-100) for 15 min each while rotating at room temperature. Washed beads were added to a 2 mL TALON Gravity Column (Clontech), and extraction buffer was added to allow the column to pack. PDI was then eluted from the packed beads using extraction buffer containing 10, 20, 40, 60, or 100 mM imidazole. Fractions were resolved by SDS-PAGE to ensure purity. PDI-containing fractions were then pooled, loaded into a 20,000 MWCO Slide-A-Lyzer Dialysis Cassette (Thermo Fisher Scientific, Waltham, MA, USA), and dialyzed with three 1 h exchanges in water. Protein concentration was calculated using the Pierce BCA Protein Assay Kit (Thermo Fisher Scientific). Purified protein was then aliquoted into 100 or 500 μg samples and allowed to freeze overnight at −80 °C before lyophilization.

### 4.3. CT Intoxication Assay

CHO cells grown to 80% confluency in 24 well plates were exposed to 10-fold dilutions of CT for 2 h in the absence or presence of 100 µM Q3R. The cells were then solubilized for 15 min at 4 °C in ice-cold acidic ethanol (1:99 HCl:EtOH) that was subsequently allowed to air dry at room temperature. Extracts were reconstituted in assay buffer for cAMP quantification using an ELISA-based kit from GE Healthcare (Piscataway, NJ, USA). Resting levels of cAMP from unintoxicated cells were background subtracted from the experimental results before presenting the data as percentages of the maximal cAMP response for the experiment.

### 4.4. CTA1 Transfection Intoxication Assay

CHO cells were transfected with pcDNA3.1/ssCTA1 [41] using a 3 h incubation with 1 µg of plasmid and LipofectAMINE (Invitrogen, Carlsbad, CA, USA) as the transfection agent. The transfected cells were then chased in medium lacking or containing 100 µM Q3R. At 4 h post-transfection, cell extracts were generated and processed for cAMP detection as described above for the CT intoxication assay. Consistent with previous reports [41], CTA1-transfected cells produced a cAMP response that was between 1.9 and 6.5-fold above the basal level of cAMP from mock-transfected cells (*n* = 4). Resting levels of cAMP from mock-transfected cells were background subtracted from the experimental results before presenting the data as percentages of the cAMP response from transfected cells chased in medium lacking Q3R.

### 4.5. SPR

A Reichert (Depew, NY, USA) dual-channel SR7000 refractometer with a flow rate of 41 µL/min was used for SPR experiments. To detect PDI binding to CTA1, His-tagged CTA1 was appended to one channel of a nickel-nitrilotriacetic acid sensor as previously described [55]. To detect PDI-driven CT disassembly, the CT holotoxin was appended to one channel of a GM1-coated SPR sensor as previously described [6]. Perfusion with phosphate-buffered saline containing 0.1% Tween-20 (PBS-T) was used to establish the baseline 0 refractive index unit (RIU) signal that corresponded to the mass of sensor-bound CTA1 or CT. PDI was then perfused over both channels in the presence of 1 mM GSH; the second channel without immobilized ligand was used as a reference cell to account for non-specific binding to the sensor. The binding assay was performed at 25 °C used a PDI concentration of 1.6 µg/mL (28 nM) with a 3000-fold molar excess of Q3R. The CT disassembly assay was performed at 37 °C and used a PDI concentration of 5.7 µg/mL (100 nM) with a 150-fold molar excess of Q3R. In both binding and disassembly assays, unbound Q3R was not removed from PDI prior to injection. Excess Q3R was instead present with PDI in the perfusion buffer. For the CT disassembly assay, sequential additions of anti-PDI (1:10,000 dilution; Enzo Life Sciences, Farmingdale, NY, USA), monoclonal 35C2 anti-CTA1 [56] (1:500 dilution), and anti-CTB (1:15,000 dilution, Sigma-Aldrich) antibodies were added to the sensor after removing PDI from the perfusion buffer. Control experiments have previously shown that PDI does not bind to the CTA2/CTB_5_ complex, that only reduced PDI binds to CTA1, and that reduction alone is not sufficient for CT disassembly [6,7,17].

### 4.6. Toxin Reduction Assay

PDI was incubated with 10 mM GSH for 30 min at room temperature before dialysis with two 1 h exchanges in 1 L PBS. An aliquot of pre-reduced PDI was then treated with a 150-fold molar excess of Q3R for 30 min at room temperature before use. To monitor toxin reduction, 1 µg of CTA was incubated at 37 °C for 30 min with 2 µg of pre-reduced PDI in a 15 µL volume. Samples were resolved by non-reducing SDS-PAGE with 15% polyacrylamide gels and visualized by Coomassie stain.

### 4.7. CTA1 Protease Sensitivity Assay

CTA was placed in 0.02 M NaPO_4_ buffer (pH 7.4) with 1 mM GSH and then aliquoted into 1 μg samples with 2 μg of PDI and/or a 150-fold molar excess of Q3R (270 µM) for a total volume of 20 μL. After 1 h at 30 °C, the samples were placed in an ice bath for 10 min and were then exposed to 0.04 mg/mL of thermolysin (final concentration) for 1 h at 4 °C. Samples were resolved by SDS-PAGE with 15% polyacrylamide gels and visualized by Coomassie stain.

### 4.8. FTIR Spectroscopy

PDI and/or uniformly ^13^C-labeled CTA1 (25 μg each) were resuspended in 75 μL of a D_2_O-based buffer containing 20 mM sodium borate buffer and 1 mM GSH. Uniformly ^13^C-labeled CTA1 was purified as previously described [6]. Samples were incubated in the absence or presence of 870 μM Q3R for 10 min before spectra were measured at room temperature using a Bruker (Madison, WI, USA) Vector 22 spectrometer. This represents a 150-fold molar excess of Q3R over PDI and a 55-fold molar excess of Q3R over CTA1. Samples containing both PDI and uniformly ^13^C-labeled CTA1 were incubated for an additional 10 min before the measurements. Data were baseline-corrected from 1700 to 1540 cm^−1^, and final figures were created with Igor Pro (Wavemetrics, Portland, OR, USA). All experiments were performed in duplicate.

### 4.9. RTB Binding Assay

Asialofetuin II (2 mg/mL in pH 9.2 sodium bicarbonate buffer) was absorbed to the wells of an ELISA plate for 18 h at 4 °C. After 4 washes with PBS-T, RTB (1 µg/mL with 2.5% BSA) was added to the plate for 1 h at 4 °C. After another 4 washes with PBS-T, PDI (20 µg/mL with 2.5% BSA) was added to the plate for 1 h at 4 °C. When indicated, PDI was treated with 1 mM DTT or a 150-fold molar excess of Q3R (60 µM) for 30 min before addition to the plate. Samples treated with both DTT and Q3R received sequential 30 min incubations with DTT and then Q3R before addition to the plate. After 4 washes with PBS-T, a rabbit anti-PDI antibody (Enzo Life Sciences, Farmingdale, NY, USA) was added to the plate for 1 h at 4 °C at 1:1000 dilution. This was followed by washing with PBS-T, a 30 min incubation at 4 °C with a 1:1000 dilution of a HRP-conjugated goat anti-rabbit IgG antibody (Jackson ImmunoResearch, West Grove, PA, USA), and further washing. Addition of TMB substrate (Thermo Fisher Scientific) for 30 min at ambient temperature, followed by the addition of a stop solution (1:5 sulfuric acid: PBS), was used for signal detection on a BioTek (Winooski, VT, USA) Synergy plate reader. To record the non-specific background signal, cells coated with asialofetuin II but not RTB were incubated with PDI, primary antibody, and HRP-conjugated secondary antibody as described above. The values recorded for these control measurements were background-subtracted from the experimental results before expressing all data as percentages of the maximal signal for the experiment.
